# “Which Factors Affect Pregnancy Until Calving and Pregnancy Loss in Buffalo Recipients of *in vitro* Produced Embryos?”

**DOI:** 10.3389/fvets.2020.577775

**Published:** 2020-12-02

**Authors:** Wilson Pardini Saliba, Lindsay Unno Gimenes, Roberti Martins Drumond, Henrique Xavier Salgado Bayão, Rossella Di Palo, Bianca Gasparrini, Marcello Rubessa, Pietro Sampaio Baruselli, José Nélio Souza Sales, Eduardo Bastianetto, Rômulo Cerqueira Leite, Mucio Túlio Teixeira Alvim

**Affiliations:** ^1^Autonomous Veterinarian, Porto Seguro, Brazil; ^2^Departamento de Patologia, Reprodução e Saúde Única, Universidade Estadual Paulista, Jaboticabal, Brazil; ^3^Cenatte Embriões Sociedade Limitada, Pedro Leopoldo, Brazil; ^4^Autonomous Veterinarian, Belo Horizonte, Brazil; ^5^Dipartimento di Medicina Veterinaria e Produzioni Animale, Università degli Studi di Napoli Federico II, Naples, Italy; ^6^Department of Animal Sciences, University of Illinois, Urbana, IL, United States; ^7^Departamento de Reprodução Animal, Universidade de São Paulo, São Paulo, Brazil; ^8^Departamento de Medicina Veterinária, Universidade Federal de Juiz de Fora, Juiz de Fora, Brazil; ^9^Departamento de Medicina Veterinária Preventiva, Universidade Federal de Minas Gerais, Belo Horizonte, Brazil; ^10^Autonomous Veterinarian, Pedro Leopoldo, Brazil

**Keywords:** embryo transfer, OPU, IVEP, fresh embryos, vitrification, embryo/fetal mortality

## Abstract

*In vitro* embryo production and embryo transfer (ET) in buffaloes has been developed for decades. However, most studies are focused on the donor or laboratory improvements, and there is a lack of reports regarding the recipients. Therefore, our aim was to investigate factors associated to pregnancy (P/ET), pregnancy loss (PL), and calving rates in buffalo recipients. The studied factors were season, recipient parity, the synchronization protocol, the CL diameter, asynchrony between the embryo and the recipient, the day of the recipient estrous cycle, the embryo (fresh vs. vitrified), the day of embryo development, and the embryo stage. These retrospective data, from a program of *in vitro* produced embryos, were analyzed by logistic regression, and the odds ratio was also estimated. Two factors were related to P/ET and the calving rate: (1) progesterone associated to estradiol plus eCG protocol for fixed time ET tended to affect positively P/ET on day 30 (41.9 vs. 36.1%, respectively; *P* = 0.07; AOR = 1.28) and P/ET on day 60 (37.8 vs. 36.1%, respectively; *P* = 0.09; AOR = 1.08) compared to the Ovsynch protocol; and (2) the CL diameter (≥14.5 mm) at transfer increased P/ET on day 30 (47.4 vs. 32.5%; *P* < 0.01; AOR = 1.87) and on day 60 (45.3 vs. 27.7%; *P* < 0.01; AOR = 2.16), and also the calving rate (37.9 vs. 21.7%; *P* < 0.01; AOR = 2.20). PL was greater when ET was done in the nonbreeding season compared to the breeding season (PL 30–60: 12.8 vs. 0.0%, *P* = 0.01; AOR > 999.99; PL 60-calving: 26.8 vs. 3.6%, *P* = 0.03; AOR = 9.90; and PL 30-calving: 36.2 vs. 3.6%, *P* = 0.01; AOR = 15.30). In conclusion, the data of our study indicated that the synchronization protocol, the CL diameter, and ET during the breeding season impacted the reproductive efficiency of buffalo recipients.

## Introduction

Brazil is home to 0.67% of the world buffalo herd, with a population of about 1,390,066 (1). Buffaloes have advantages due to their rusticity, adaptability, and the quality of their main products, the milk, and the meat.

Several reproductive biotechnologies, such as superovulation for *in vivo* embryo production (SOV) or ovum pick-up (OPU) for *in vitro* embryo production (IVEP), have been used in order to obtain animals with better performance and high genetic merit (2). Unfortunately, the efficiency of superovulation for *in vivo* embryo transfer (ET) in buffaloes is low when compared to cattle (3). For this reason, the association of OPU and IVEP is currently the most competitive and suitable solution for this purpose, reducing the interval between generations (3, 4).

The strategy of embryo transfer (ET) has enabled genetic progress through maternal lineage and also contributed to the control of infectious diseases (5). Besides the use of fresh embryos, both conventional freezing and vitrification were used for cryopreservation, resulting in pregnancy and calving (6–12), although low pregnancy/embryo transfer [P/ET; 10.5–26.9% (8, 10, 11)] and calving rates [10.0–25% (8, 9, 11, 12)] were generally reported.

In cattle, the recipient plays a critical role in the success of ET programs (13, 14). Several factors are associated with P/ET and calving rates, such as the diameter of corpus luteum (CL), synchronization protocols, season (14), the synchrony between the embryo stage and the recipient, embryo quality, and recipient management (15). However, in buffaloes, only one retrospective study regarding recipients was carried out (16). In that article, asynchrony >12 h and the use of grade I embryos were the factors that enhanced P/ET; the CL diameter also tended to increase P/ET. Since then, few studies were focused on buffalo recipients and, to our knowledge, none were conducted to evaluate multiple factors influencing pregnancy and calving outcomes or pregnancy loss after ET using *in vitro* produced embryos.

Therefore, the aim of the present study was a retrospective analysis of a dataset obtained from an ET program in order to determine which factors can affect pregnancy, calving rates, and pregnancy loss in buffalo recipients.

## Materials and Methods

### Experimental Location and Animals

The dataset was obtained from 201 ovum pick-up (OPU) sessions and 184 ETs carried out at Cenatte Embriões LTDA, located in the city of Pedro Leopoldo, Minas Gerais, Brazil (latitude: 19°37′12″ south, longitude: 44°2′38″ west).

A total of 73 crossbred Murrah buffalo females (23 nulliparous and 50 multiparous), healthy, nonpregnant, and cycling were used as oocyte donors and embryo recipients. The animals were kept under grazing of *Brachiaria decumbens* and *B. brizantha* with free access to water and mineral salt. During the autumn and winter periods, all animals were supplemented with elephant grass silage to meet the demand of each category (17).

### Management of Oocyte Donors

All females were used as oocyte donors along the experimental period, and groups of 6–36 animals were submitted to 11 replicates with a minimum interval of 19 days, for 8 months, without any hormonal stimulation.

The animals were contained and submitted to epidural anesthesia, using 3 ml of 2% lidocaine (Lidovet®, Bravet). OPU procedure was performed using transvaginal ultrasound equipment (Aloka 500®, Tokyo, Japan; frequency 5 MHz) coupled to a system with an 18G disposable hypodermic needle (BD) and a 1.7-mm-diameter and 80-cm-long Teflon aspiration line (WTA, Watanabe Applied Technology). The vacuum pressure used was 60–65 mmHg (10 and 12 ml of water/minute). A warm solution (37°C) containing PBS with sodium heparin (5,000 UI/L; Parinex®, Hypolabor), 1% fetal bovine serum (FBS, Nutricell), and 0.1% antibiotic solution (amikacin) was used to collect the aspirated oocytes from follicles larger than 2 mm.

After OPU, cumulus-oocyte complexes (COCs) were searched and classified under a stereomicroscope. TCM 199 with 5% FBS, pyruvate (0.2 mM), and amikacin (83.4 mg/ mL) was used to wash the COCs collected. The criteria used for the evaluation of COC were the presence, the number of layers, and the degree of compaction of *Cumulus* cells, as well as the appearance of the cytoplasm in color, homogeneity, and integrity. Up to their parameters, the COCs were classified as follows: grade I (three or more layers of cells of the compact cumulus and homogeneous cytoplasm), II (one to three layers of cumulus cells covering totally or partially the oocyte and with homogeneous or heterogeneous cytoplasm), and III (one layer of cumulus cells covering the oocyte totally or partially and with cytoplasm homogeneous or heterogeneous), denuded, degenerated, expanded, and atretic (18). For *in vitro* embryo production (IVEP), only oocytes grades I, II, and III (viable oocytes) were used.

Viable oocytes were maintained in 1.2 ml cryovials (Corning) containing an oocyte maturation medium, inside an incubator for embryo transport (WTA) at 38.5°C with gas mixture (5.5% CO_2_, 5.0% O_2_, and 89.5% N_2_), until the arrival to the laboratory. The maximum transport time to the laboratory was 6 h after the beginning of OPU.

### *In vitro* Embryo Production (IVEP)

In the laboratory, groups of 10 to 12 oocytes were transferred to 50-μL drops of a maturation medium, composed of TCM199 with sodium bicarbonate supplemented with 10% FBS, pyruvate (0.2 mM), amikacin (83.4 mg/ mL), 0.5 μg of FSH (Folltropin), 5 μg of LH (Lutropin), 1 μg of 17 α-estradiol, and supplemented with 50 μM of 2-mercaptoethanol, covered with sterile mineral oil. Oocytes were incubated for 20–24 h, at 38.7°C, with 5.5% CO_2_ in air and saturated humidity.

One hour before the end of the maturation period, the semen from a tested bull was thawed at 35°C for 20 s and deposited on a Percoll gradient (30, 60, and 90%) for sperm selection. Centrifugation was performed at 9,000 × g for 2 min, and, subsequently, the pellet was washed twice in a total alkaline phosphatase medium (TALP), at 4,500 × g for 1 min. The inseminating dose was adjusted to 1.2 million sperm/mL. After IVM, the oocytes were washed in a TCM199 HEPES medium containing 0.3% BSA, pyruvate (0.2 mM), and amikacin (83.4 mg/mL). Then they were washed in a TALP fertilization medium with 0.5% BSA, pyruvate (0.2 mM), amikacin (83.4 mg/ mL), heparin (20 μg/ mL), and PHE solution (2 μM of penicillamine, 1 μM of hypotaurine, and 0.25 μM of epinephrine), and groups of 10–12 oocytes were co-cultured with sperm in 50 μL drops of this same medium, under the same conditions of atmosphere, humidity, and temperature used for IVM for 18–20 h.

After fertilization (D0), the presumptive zygotes were denuded, and groups of 10–12 were transferred to 100 μL drops containing a SOFaa medium with 3% FBS under mineral oil, during 5–7 days at 38.7°C, with 5.5% CO_2_, 5.0% O_2_, and 89.5% N_2_ in saturated humidity. Forty-eight hours after fertilization, the embryo cleavage was evaluated. There was no change of medium throughout the *in vitro* culture. On 5th to 7th days after fertilization (D5–D7, respectively), the blastocyst rate (blastocysts/viable oocytes) was calculated.

### Embryo Evaluation and Preparing for Fresh or Vitrified Transfer

Embryos were morphologically evaluated and classified according to the IETS manual (19), in terms of developmental stage: early blastocyst, blastocyst, expanded blastocyst, and hatched blastocyst; and quality: grade I (excellent or good), II (fair), III (poor), or IV (degenerated).

Only grade I D5 and D6 embryos, varying from early to hatched blastocysts, were transferred. The embryos were loaded in 0.25-mL straws containing a maturation washing medium (TCM 199 HEPES, 5% FBS, pyruvate, and amikacin) and were maintained in the portable incubator until transfer to recipients.

Vitrification was performed when the number of embryos exceeded the number of recipients. Only grade I D5, D6, and D7 embryos, varying from expanded to hatched blastocysts, were vitrified on Cryotop® according to the method described by De Rosa et al. (20). Briefly, the embryos were placed in a solution containing 7.5% ethylene glycol and 7.5% dimethyl sulfoxide (DMSO), prepared in TCM 199 HEPES supplemented with 20% FBS, for 3 min and then were transferred to a second solution containing 16.5% ethylene glycol, 16.5% DMSO prepared in FBS with 0.5 M sucrose. After 25 s, the embryos were kept in a volume of <0.1 μL of vitrification solution and loaded on Cryotop®, which was immediately immersed in liquid nitrogen. The warming procedure was carried out by immersing the top of Cryotop® containing the embryos in a 0.25 M sucrose solution. After 1 min, the embryos were transferred to a 0.15 M sucrose solution for 5 min and then loaded in 0.25-mL straws for transfer to recipients.

### Recipients Management

All females previously used as oocyte donors were also used as recipients, along 24 months, totalizing 184 procedures divided in 18 replicates (fresh: *n* = 10; vitrified: *n* = 8 replicates).

All recipients were submitted to one of the two following synchronization protocols: Ovsynch (21) or estradiol plus progesterone based (22). Briefly, the Ovsynch protocol consisted of 0.5 mg of lecirelin (Gestran Plus®, Tecnopec) on a random day of estrous cycle. Seven days later, 0.5 mg of cloprostenol (Sincrosin®, MSD Animal Health) was administered. A second dose of lecirelin (0.25 mg) was injected 48 h later. The second protocol consisted in the insertion of an intravaginal progesterone device, used for 8 days (multiparous) or 16 days (nulliparous) associated to 2 mg of estradiol benzoate (Estrogin®, Biofarm) and 530 mg of cloprostenol, on a random day of estrous cycle. Nine days later, the devices were removed and 400 IU of eCG (Folligon®, MSD Animal Health) and PGF2α were administered to all recipients. Finally, after 48 h, the recipients were treated with 0.5 mg of lecirelin (Gestran Plus®, Tecnopec).

Six or seven days later, ovulation was evaluated by the presence of a CL, which was measured by ultrasound (Aloka 500®, Tokyo, Japan; frequency 5 MHz), and fresh or vitrified embryos were transferred in the uterine horn ipsilateral to CL, by a nonsurgical method. Only one embryo was transferred in each recipient. Asynchrony between embryos and recipients was ±1 day.

### Monitoring of Pregnancy and Birth

Pregnancies were monitored by ultrasound (Aloka 500, Tokyo, Japan; frequency 5 MHz) 30 and 60 days after the expected ovulation. At the due day, the animals were evaluated for labor stages, dystocia, and placental retention. The newborns were weighed, the sex determined, and suckling reflex and umbilicus were examined.

P/ET was calculated as the percentage of pregnant females, diagnosed on days 30 (P/ET 30) and 60 (P/ET 60), on the total number of the recipients. The calving rate was calculated as the percentage of females calving out on the total number of recipients. PL was calculated as the percentage of nonpregnant females, from days 30 to 60, day 60 to calving, and day 30 to calving, in relation to females pregnant on days 30, 60, and 30, respectively.

### Statistical Analysis

Data concerning *in vitro* embryo production were shown according to descriptive statistics, except by analysis of birth weight associated to sex of calves, which was analyzed by logistic regression, by using the GLIMMIX procedure in SAS for Windows (23). Data regarding recipients were analyzed by univariate and multivariate logistic regression, using the LOGISTIC procedure (23). All groups with fewer than 10 recipients were excluded from the analysis. For pregnancy and calving rate analysis, the following were excluded: asynchrony (−1 day, *n* = 7), day of embryo development (day 7, *n* = 7), and embryo stage (hatched blastocyst, *n* = 9). For pregnancy loss, the following were excluded: asynchrony (−1 day, *n* = 2), day of embryo development (day 7, *n* = 2), and embryo stage (early blastocyst, *n* = 5; blastocyst, *n* = 8; hatched blastocyst, *n* = 5). Adjusted odds ratio (AOR) and 95% confidence interval (CI) were generated by logistic regression. Odds ratio is a measure of association between a treatment and an outcome and represents the odds that an outcome will occur given a particular treatment compared to the odds of the outcome occurring in the absence of that treatment or another treatment (24). Results are presented as percentages and AOR. *P* ≤ 0.05 were considered significant, and those 0.05 ≤ *P* ≤ 0.10 were considered as tendency.

## Results

### Overall Embryo Rate, P/ET, Calving Rates, and PL

A total of 998 oocytes were recovered from 201 OPU sessions (5.0 ± 0.5/donor), of which 584 were viable oocytes (2.9 ± 0.3/donor). A total of 318 structures cleaved (1.6 ± 0.2/donor), resulting in a cleavage rate of 54.5%. The blastocyst rate was 44.9% producing 262 embryos (1.3 ± 0.1/donor), of which 229 were grade I, 5 grade II, and 28 grade III.

From the total embryos produced, 114 were transferred fresh resulting in P/ET 30, P/ET 60, and a calving rate of 43.0% (49/114), 41.2% (47/114), and 35.1% (40/114), respectively. Pregnancy loss between days 30 and 60 was 4.1% (2/49), between day 60 and calving was 14.9% (7/47), and between day 30 and calving was 18.4% (9/49). The mean gestation length was 310.2 ± 0.5 days. All calvings were eutocic, and the average weight of calves born was 32.6 ± 0.2 kg, being lower for females (31.4 ± 0.4 kg; *n* = 20) than for males (33.8 ± 0.6 kg; *n* = 20; *P* < 0.01). Concerning postpartum occurrences, two females showed retained placenta (5%), two cervix prolapse (5%), and one rejected her calf (2.5%).

For vitrified embryos, 70 were transferred and P/ET 30, P/ET 60, and the calving rate were, respectively, 37.1% (26/70), 31.4% (22/70), and 24.3% (17/70). Pregnancy loss between days 30 and 60 was 15.4% (4/26), between day 60 and calving was 22.7% (5/22), and between day 30 and calving was 34.6% (9/26). The mean gestation length was 314.5 ± 0.9 days, and one of the animals presented dystocia. The average weight of calves born was 33.8 ± 0.2 kg, being similar in females (33.6 ± 0.7 kg; *n* = 10) and males (34.0 ± 0.4 kg; *n* = 7; *P* = 0.33). In the postpartum period, retained placenta was not observed in any recipient, and one of the calves had suckling difficulty in the first 24 h (5.9 %).

### Factors Associated to Pregnancy and Calving Rate in Buffalo Recipients

Pregnancy and calving rates were significantly associated to the synchronization protocol and the CL diameter. Reproductive season, parity, asynchrony, day of recipient estrus, type of embryo, and day or stage of embryo development did not affect P/ET 30, P/ET 60, or the calving rate (Table 1).

**Table 1:** Factors associated to pregnancy (P/ET) on days 30 and 60, and calving rate in buffalo recipients.

		**P/ET**				**Calving rate**	
	***N***	**30 days**	OR (95% CI)^*^	**60 days**	**OR (95% CI)**	**Calving**	**OR (95% CI)**
**Season**							
Breeding season	80	35.0	Ref.^#^	35.0	Ref.	33.8	Ref.
Nonbreeding season	104	45.2	1.53 (0.84–2.79)	39.4	1.21 (0.66–2.21)	28.9	0.80 (0.43–1.49)
**Recipient parity**							
Cow	134	40.3	Ref.	36.6	Ref.	28.4	Ref.
Heifer	49	40.8	1.02 (0.56–1.99)	38.8	1.10 (0.56–2.16)	36.7	1.47 (0.74–2.93)
**Protocol**							
Ovsynch	36	**36.1**^**b**^	Ref.	**36.1**^**b**^	Ref.	33.3	Ref.
P4 + EB^$^	148	**41.9**^**a**^	1.28 (0.6–2.71)	**37.8**^**a**^	1.08 (0.51–2.30)	30.4	0.87 (0.40–1.90)
**CL diameter**							
<14.5 mm	83	**32.5**^**B**^	Ref.	**27.7**^**B**^	Ref.	**21.7**^**B**^	Ref.
≥14.5 mm	95	**47.4**^**A**^	1.87 (1.01–3.44)	**45.3**^**A**^	2.16 (1.15–4.04)	**37.9**^**A**^	2.20 (1.13–4.29)
**Asynchrony**							
0	47	34.0	Ref.	29.8	Ref.	21.3	Ref.
+1	122	42.6	1.44 (0.71–2.90)	40.2	1.58 (0.77–3.26)	34.4	1.94 (0.88–4.29)
**Day of recipient estrous cycle**							
7	73	41.1	Ref.	38.4	Ref.	31.5	Ref.
6	111	40.5	0.98 (0.54–1.78)	36.9	0.94 (0.51–1.73)	30.6	0.96 (0.51–1.82)
**Embryo**							
Vitrified	70	37.1	Ref.	31.4	Ref.	24.3	Ref.
Fresh	114	43.0	1.28 (0.69–2.35)	41.2	1.53 (0.82–2.87)	35.1	1.69 (0.86–3.29)
**Day of embryo development**							
6	114	36.5	Ref.	33.1	Ref.	26.1	Ref.
5	55	48.2	1.61 (0.84–3.11)	46.3	1.75 (0.90–3.38)	40.7	1.95 (0.98–3.86)
**Embryo stage**							
Expanded blastocyst	135	40.7	Ref.	37.8	Ref.	30.4	Ref.
Blastocyst	18	44.4	1.16 (0.43–3.14)	38.9	1.05 (0.38–2.88)	38.9	1.46 (0.53–4.03)
Early blastocyst	15	33.3	0.73 (0.24–2.25)	33.3	0.82 (0.27–2.55)	26.7	0.83 (0.25–2.77)

* *OR, odds ratio; CI, confidence interval*.

# *Ref., reference point odds ratio = 1.0*.

$ *P4 + EB: progesterone associated to estradiol plus eCG protocol*.

*A ≠ B (P ≤ 0.05); a ≠ b (0.05 ≤ P ≤ 0.10)*.

When progesterone associated to estradiol plus the eCG-based protocol was compared to Ovsynch, there was a tendency of greater P/ET 30 (41.9 vs. 36.1%; *P* = 0.07; AOR = 1.28) and P/ET 60 (37.8 vs. 36.1%; *P* = 0.09; AOR = 1.08).

Recipients with a CL diameter larger than or equal to 14.5 mm had increased P/ET 30 (47.4 vs. 32.5%; *P* < 0.01; AOR = 1.87), P/ET 60 (45.3 vs. 27.7%; *P* < 0.01; AOR = 2.16), and a calving rate (37.9 vs. 21.7%; *P* < 0.01; AOR = 2.20), when compared to those with CL <14.5 mm.

### Factors Associated to Pregnancy Loss in Buffalo Recipients

Pregnancy loss (PL) was associated only to the season of the embryo transfer. No other factor influenced PL from days 30 to 60 (PL 30–60), day 60 to calving (PL 60-C), or day 30 to calving (PL 30-C; Table 2).

**Table 2:** Factors associated to pregnancy loss (PL) from days 30 to 60, day 60 to calving, and day 30 to calving in buffalo recipients.

		**PL**					
	***N***	**Days 30–60**	OR (95% CI)^*^	**60 days–calving**	**OR (95% CI)**	**30 days–calving**	**OR (95% CI)**
**Season**							
Breeding season	28	**0.0**^**A**^	Ref.^#^	**3.6**^**A**^	Ref.	**3.6**^**A**^	Ref.
Nonbreeding season	47	**12.8**^**B**^	>999.90 (<0.01–∞)	**26.8**^**B**^	9.90 (1.20–81.80)	**36.2**^**B**^	15.30 (1.91–122.80)
**Recipient parity**							
Heifer	20	5.0	Ref.	5.3	Ref.	10.0	Ref.
Cow	54	9.3	1.93 (0.21–17.70)	22.4	5.21 (0.62–43.52)	29.6	3.79 (0.79–18.27)
**Protocol**							
Ovsynch	13	0.0	Ref.	7.7	Ref.	7.7	Ref.
P4 + EB^$^	62	9.7	>999.90 (<0.01–∞)	19.6	2.93 (0.34–25.02)	27.4	4.53 (0.55–37.58)
**CL diameter**							
≥14.5 mm	45	4.4	Ref.	16.3	Ref.	20.0	Ref.
<14.5 mm	27	14.8	3.74 (0.64–21.97)	21.7	1.43 (0.39–5.14)	33.3	2.00 (0.68–5.91)
**Asynchrony**							
0	16	12.5	Ref.	28.6	Ref.	37.5	Ref.
+1	52	5.8	0.43 (0.07–2.83)	14.3	0.42 (0.10–1.71)	19.2	0.40 (0.12–1.35)
**Day of recipient estrous cycle**							
7	30	6.7	Ref.	17.9	Ref.	23.3	Ref.
6	45	8.9	1.37 (0.23–7.97)	17.1	0.95 (0.27–3.35)	24.4	1.06 (0.36–3.14)
**Embryo**							
Fresh	49	4.1	Ref.	14.9	Ref.	18.4	Ref.
Vitrified	26	15.4	4.27 (0.73–25.12)	22.7	1.68 (0.47–6.05)	34.6	2.35 (0.79–6.96)
**Day of embryo development**							
5	26	3.9	Ref.	12.0	Ref.	15.4	Ref.
6	42	9.5	2.6 (0.28–24.93)	21.1	2.0 (0.47–8.22)	28.6	2.2 (0.63–7.74)
**Embryo stage**							
Expanded blastocyst	57	8.8	–	19.2	–	26.3	–

* *OR, odds ratio; CI, confidence interval*.

# *Ref.: reference point odds ratio = 1.0*.

$ *P4 + EB: progesterone associated to estradiol plus eCG protocol*.

*∞ > 999.99*.

*A ≠ B (P ≤ 0.05)*.

PL was greater when ET was performed in the nonbreeding season compared to the breeding season (PL 30–60: 12.8 vs. 0.0%, *P* = 0.01; AOR > 999.99; PL 60-C: 26.8 vs. 3.6%, *P* = 0.03; AOR = 9.90; and PL 30-C: 36.2 vs. 3.6%, *P* = 0.01; AOR = 15.30).

## Discussion

The interest in large-scale production of buffalo embryos has been growing worldwide (3). Despite this, embryo recipients are still neglected compared to donors of oocytes and embryos, and few studies were conducted to clarify aspects related to pregnancy and even fewer to clarify aspects related to calving and pregnancy loss. To our knowledge, the present retrospective nonrandomized study is the first to achieve this purpose for buffalo recipients.

In this study, the CL diameter was the main factor that influenced pregnancy until calving. Although controversial, several studies in cattle demonstrated that P/ET was higher when recipients had larger CL, mainly due to an increase in progesterone levels (14). High levels of progesterone allow embryo elongation and increase interferon τ production by the conceptus, avoiding luteolysis in the critical period of maternal recognition of pregnancy (25). In buffaloes, few trials were conducted to evaluate these features. In a study with synchronization of ovulation protocol for fixed time artificial insemination (AI) in buffaloes, a significant and positive correlation between the diameter of dominant follicle at AI and the CL diameter 10 days later, ovulation rate, and pregnancy/AI was found, besides a negative correlation with embryo mortality (22). In another trial (26), cows categorized with a larger diameter of pre-ovulatory follicles on the day of estrus had a positive correlation with the CL diameter and progesterone concentration, and these variables were greater in pregnant than in nonpregnant females. Interestingly, in that work, nonpregnant females had CL diameters close to 15 mm (and progesterone <1 ng/ mL), corroborating Di Palo et al. [1990, revised in (27)] who reported higher fertility in buffaloes with CL ≥ 15 mm. Therefore, the data of the present study support that the CL diameter (≥14.5 mm) can be used as a parameter to select the recipients in buffaloes in order to obtain better outcomes.

Another finding associated to P/ET 30 and P/ET 60 was the synchronization protocol. The Ovsynch protocol has been used in buffaloes to synchronize ovulation for fixed time artificial insemination (21). As previously shown in buffaloes and cattle, the main disadvantage of this protocol is the response to the first GnRH, which can depend on the status of the dominant follicle in the beginning of treatment (28–30). Nevertheless, in anestrus animals, the efficiency of this protocol results in low follicular response and conception rates (21). To overcome this negative effect, treatments based on the association of progesterone and estradiol esters, like in cattle, have been also applied to buffalo reproductive management, with positive pregnancy results independent of animal cyclicity (22). Besides that, in the present study, eCG was associated to P4 plus the E2-based protocol. The beneficial effect of eCG on the maximum diameter of the dominant follicle, the ovulation rate, the CL diameter, CL development, and progesterone production is well-documented in cattle and buffaloes, culminating with higher P/ET (14, 31, 32).

Concerning the PL, our data demonstrated an association between PL and the season when the embryos were transferred. When ET occurred in the nonbreeding season (from October to April), a consistent embryo and fetal mortality was observed along all periods analyzed. A seasonal effect on late embryo mortality (between 26 and 40 days of pregnancy) was previously described by Campanile et al. (33). The authors recorded 45% embryo mortality when the buffaloes were inseminated during periods of increasing daylight. These authors explained this result with a reduced CL capacity to secrete progesterone. Moreover, Vecchio et al. (34) reported high incidence of fetal mortality (between 45 and 90 days of pregnancy) in buffaloes naturally mated in increasing daylight. Overall, these works confirmed our data and indicate that new trials should be performed in order to overcome early and late PL in buffaloes when reproductive strategies are conducted during the nonbreeding season.

**Figure 1:**
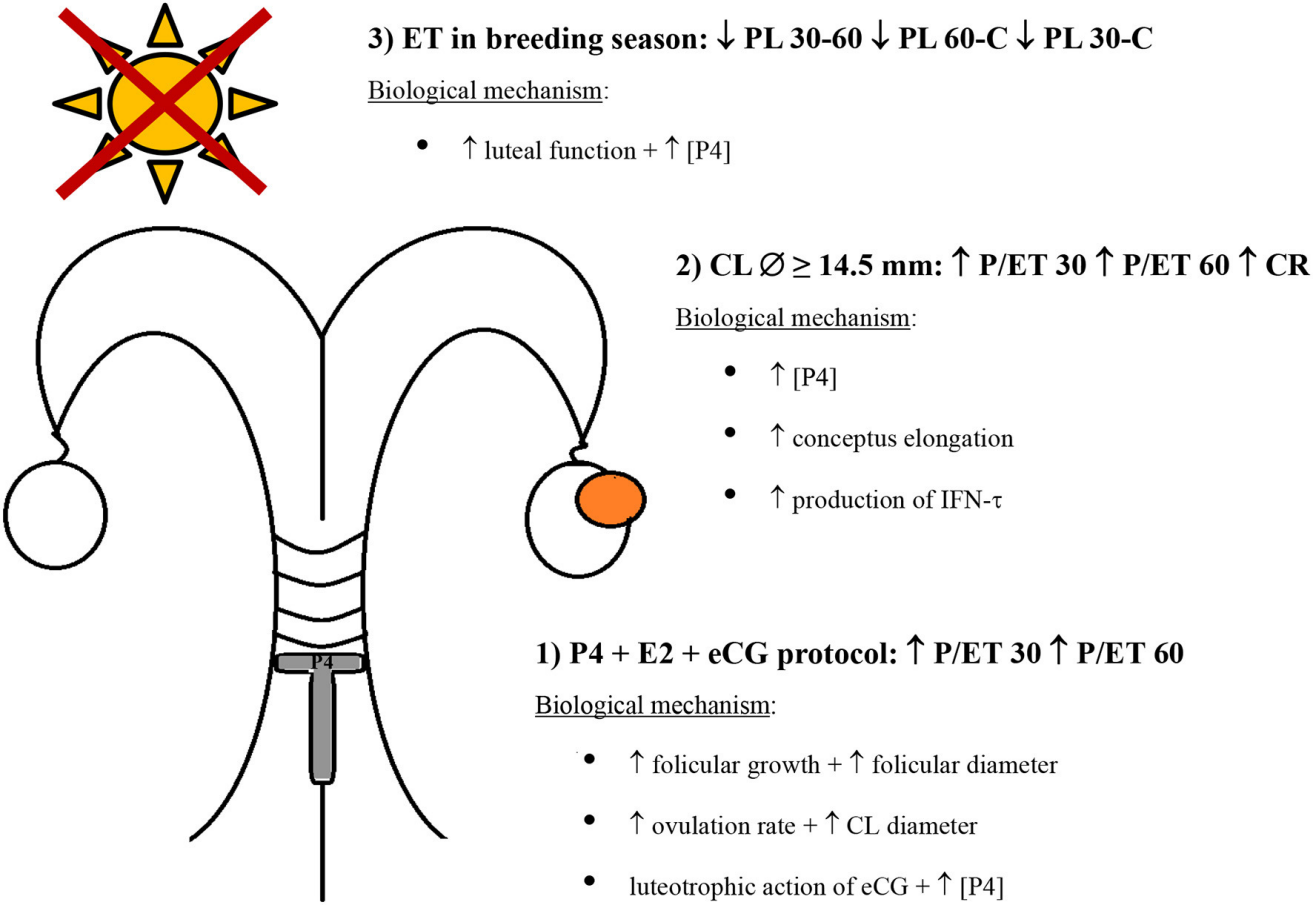
Graphic model of main factors affecting pregnancy per embryo transfer on day 30 (P/ET30), on day 60 (P/ET60), the calving rate (CR), and pregnancy loss from day 30 to 60 (PL 30-60), from day 60 to calving (PL 60-C), and from day 30 to calving (PL 30-C) in buffalo recipients of embryos produced *in vitro*. (1) Effect of the synchronization protocol on P/ET (P4 vs. Ovsynch), (2) effect of the CL diameter on P/ET (≥14.5 vs. <14.5 mm), and (3) effect of season of year on pregnancy loss (breeding season vs. nonbreeding season).

Although in the present study progesterone levels were not analyzed, the factors affecting P/ET, the calving rate, and PL herein found collectively suggest that strategies that favor the increase in progesterone levels (synchronization protocols based on progesterone associated to estradiol plus eCG, selection of recipients by the CL diameter, and ET during the breeding season) can improve P/ET until calving and reduce embryo/fetal mortality (Figure 1).

Regarding the other factors analyzed, parity did not influence the outcomes of buffalo recipients in the present study, in accordance to another work (35) in which P/ET and pregnancy per treated animal did not differ between nulliparous, primiparous, or multiparous buffaloes submitted to a protocol of fixed time ET. This reinforces the optimization of different animal categories in ET programs in buffaloes.

Misra et al. (16) reported that asynchrony ≥ 12 h impaired pregnancy rates in buffalo recipients. Inversely, our data support the possibility of asynchrony between the recipient and the embryo of 1 day (0 and +1) without negative effects on the pregnancy of buffalo recipients, as observed in cattle in which ±24 h did not affect pregnancy rates (36). Associated with this outcome, the day of recipient estrous cycle and the day or stage of embryo development also did not influence our study, in accordance with trials performed in cattle (36, 37) and in buffalo (8, 16). Collectively, the absence of effect of these factors indicates a possible flexibilization in ET schedule allowing the widespread use of this technique in buffalo farms.

In the present study P/ET, the calving rate, or PL was not affected by the type of embryo (fresh vs. vitrified). In cattle, the transfer of fresh embryos is associated to better pregnancy results and lower PL than the transfer of frozen-thawed embryos (36, 37). This can be attributed to injuries caused during cryopreservation. However, in buffaloes, studies comparing these different embryos in contemporary conditions were not found. Nevertheless, our results evidence that vitrification is efficient in buffaloes and can be used to optimize ET programs in this species.

*In vitro* embryo production outcomes in the current study were, in general, above the average reported in the literature. In previous studies, a low number of total oocytes [1.2 ± 0.07 to 4.1 ± 0.5 (38, 39)], viable oocytes [1.2 ± 0.2 to 1.9 ± 0.3 (38, 40)], blastocyst rate [10–30% (4, 41–43)], P/ET (0–26.9%), and calving rate for fresh [12.5–25.5% (6, 9, 12)] and vitrified embryos [P/ET: 10.5–16.4%, and 10–10.9%, respectively (8, 10, 11)] were reported. These differences can be attributed to several factors, including genetics, nutrition, environment, individual variation in the number of follicles/oocytes, the criterion of oocyte selection, oocyte quality, culture media, use of antioxidants, atmospheric gas, and sire, among others. Corroborating the current study, similar PL [20–33.3% (8, 11)], gestation length [302.3–319.7 days (11, 12)], and weight of calves [27–38.8 kg (8, 10, 12)] were observed. In the present study, no occurrences of large calf syndrome, hydramnio, hydroalantoid, or umbilical cord anomalies were detected. For abnormal postpartum incidence (retained placenta, cervix prolapse, calf rejection, and difficulty of suckling), 12.5 and 5.9% for fresh and vitrified embryos, respectively, were found in the present study. However, no information regarding postpartum problems in buffalo recipients was found in the literature.

## Conclusion

Our findings indicate that strategies applied to recipients, as synchronization protocols based on progesterone associated to estradiol plus eCG, selection of recipients by the CL diameter, and ET during breeding season, can improve P/ET and calving rates, reducing PL in buffaloes. However, further prospective studies with a larger number of recipients are necessary to confirm these findings.

## Data Availability Statement

The datasets generated for this study are available on request to the corresponding author.

## Ethics Statement

The animal study was conducted according to all ethical care in animal use, according to law number 11,794/ 2008 from Brazilian Constitution (Arouca Law).

## Author Contributions

WS and MA conceived the study. RM, HB, WS, LG, PB, RD, BG, MR, EB, and RL conducted the experiment. JS analyzed the data. LG wrote the manuscript. PB, BG, RD, MR, and EB critically revised the manuscript. All authors approved the final version of the manuscript.

## Conflict of Interest

The authors declare that the research was conducted in the absence of any commercial or financial relationships that could be construed as a potential conflict of interest. The handling editor declared a past co-authorship with the authors BG and PB.
